# “Treated as second class citizens” - the lived experience of obesity-related stigma: an IMI2 SOPHIA study

**DOI:** 10.1080/17482631.2024.2344232

**Published:** 2024-04-25

**Authors:** Eva Hollmann, Emma Farrell, Carel Le Roux, Joe Nadglowski, Deirdre McGillicuddy

**Affiliations:** a School of Education, University College Dublin, Belfield, Dublin, Ireland; b Diabetes Complications Research Centre, University College Dublin, Dublin, Ireland; c Governance and Financials, President/CEO Obesity Action Coalition, Tampa, FL, USA; d School of Education, University College Dublin, Dublin, Ireland

**Keywords:** Obesity, stigma, photovoice, lived experience, qualitative interviews

## Abstract

**Purpose:**

Obesity-related stigma impacts on and shapes the physical and psychosocial wellbeing of individuals living with obesity. Often absent from the literature in the field is the voice(s) of those living with obesity capturing the nuances of the lived experiences of obesity-related stigma.

**Methods:**

This study adopted a qualitative approach encompassing individual (*n* = 15) and photovoice method (*n* = 12), with a purposeful sample of patients accessing treatment for obesity within the healthcare setting during 2021. Analysis was undertaken using thematic analysis.

**Results:**

Key themes developed from the analysis related to experiencing obesity-related stigma as exposure to external judgement, societal exclusion and felt environmental stigmatization. Exposure to external judgement was described as judgemental comments resulting in hypervigilance to societal judgement. Participants reported how being overlooked and ignored by others had various negative effects and compounded obesity-related stigma through societal exclusion. Public spaces lacking suitable equipment further made obesity-related stigma visible through felt environmental stigmatization when pursuing hobbies and in everyday life.

**Conclusions:**

Obesity-related stigma had a profoundly negative impact on participants in this study, particularly in shaping social interaction, limiting life experiences and impacting psychosocial wellbeing.

## Introduction

Obesity as a disease presents a global threat due to its negative impact on people’s health as well as an “increasing prevalence globally” (Fruh, 2017, p. 511). Obesity is defined as an “abnormal or excessive fat accumulation that presents a risk to health” (WHO, 2022). It was recognized by the European Parliament’s Committee on the Environment, Public Health and Food Safety in 2020 as a chronic disease (EASO, 2020). Chronic diseases are defined “broadly as conditions that last 1 year or more and require ongoing medical attention or limit activities of daily living or both” (NCCDPHP, 2022). Most recent figures show that in 2035, 47% of adults of the population in the Ireland will be living with obesity (World Obesity Federation, 2023). Globally, 650 million people were living with obesity in 2016 (WHO, 2021). Between 1980 and 2015, the socio-demographic index of a country was proportional to its obesity prevalence (The GBD 2015 Obesity Collaborators, 2017). In Ireland, the Healthy Ireland Survey 2015 suggested that 23% of participants, aged 15 and older, were living with obesity in 2015 (Ipsos MRBI, 2015). Obesity can increase the risk of mortality and lead to other chronic diseases, such as cardiovascular diseases, cancer, type 2 diabetes, obstructive sleep apnoea and psychological conditions, which can further impact quality of life (Fruh, 2017; Hruby et al., 2016; Taylor et al., 2013).

Goffman (2009) defined stigma as an individual’s failure to fit into a preassigned category due to an unfavourable characteristic which leads to a perception of not fitting into the norm. The category allocated to a person depends on their social identity. This identity consists of the personal and structural characteristics which are associated with the individual. If people meet a person who does not quite fit into these groupings, they start identifying their social identity. If the individual, instead of showing a feature that fits into one of the categories, provides an unfavourable attribute, the individual is diminished to a person that is not normal. This feature can be seen to represent stigma (Goffman, 2009).

People with obesity have been identified as experiencing obesity-related stigma (Kim et al., 2019; Lewis et al., 2011). Enacted stigma describes a form of external obesity-related stigma, which is experienced as negative, discriminatory, presumptuous or judgemental behaviour by people who are living with obesity due to their physique (Prunty et al., 2020; R. Puhl & Brownell, 2001; R. M. Puhl & Heuer, 2009; R. Puhl & Suh, 2015). Felt stigma, on the other hand, relates to the concern of not being socially accepted (Scambler, 1998). Prunty et al. (2020) identified that 57% of participants in their study who were living with obesity reported instances of enacted stigma. Vartanian et al. (2014) found that the average of people with obesity who were included in their study encounter obesity-related stigma about 11 times within a “two-week period” (Vartanian et al., 2014, p. 200). Stigma can be experienced as direct (stigmatizing behaviour), environmental (such as lack of appropriate equipment) or indirect (fear of being judged or discriminated against) (Lewis et al., 2011). The insidiousness of such obesity-related stigma experiences has been reported in all aspects of individual’s lives from family, friends and partners (The GBD 2015 Obesity Collaborators, 2016; Vartanian et al., 2014), to public settings (Tapking et al., 2020), and within the healthcare system (Flint, 2015; Malterud & Ulriksen, 2011; Tapking et al., 2020). A scoping review conducted by de MacêDo et al. (2022) has found that obesity-related stigma was present in areas of everyday life such as the media and during interaction with other people during the COVID-19 pandemic. The negative attributes used to enact stigma (Goffman, 2009) and ascribed specifically to people living with obesity are often characterized by characteristics such as “laziness” and “inactivity” (Tapking et al., 2020, p. 4931).

Obesity-related stigma has resulted in a counterproductive outcome hindering the reduction in obesity case numbers overall (Flint, 2015). Previous research has also identified the link between obesity-related stigma and psychological wellbeing (Jackson, 2016). With negative impacts on mental health manifest through psychological distress such as depression (Alimoradi et al., 2020) and impacting on quality of life measures (R. Puhl & Suh, 2015). A qualitative synthesis of studies identified that people with obesity experience stigma, shame, judgement and blame (Farrell et al., 2021). The literature also suggests that the lived experience of living with obesity is characterized by discrimination, societal exclusion (Thomas et al., 2008) and “objectification and alienation” from oneself and others (Ueland et al., 2019, p. 9).

This study was conducted as part of the IMI2 SOPHIA (Stratification of Obesity Phenotypes to Optimize Future Treatment) project.^1^

While the literature provides us with insights into obesity-related stigma (Asbury & Woszidlo, 2016; Farrell et al., 2021; Flint, 2015; Malterud & Ulriksen, 2011; Tapking et al., 2020; Thomas et al., 2008; Ueland et al., 2019; Vartanian et al., 2014), identifying nuances of the lived experience of the phenomenon in an Irish context can play an important role in understanding the issue of obesity-related stigma. This paper presents findings from a qualitative study exploring the lived experience of living with obesity as describe in-depth by participants through conversational interviews, photographs capturing and reflecting their stories and group discussions to share and reflect on the experience of living with obesity. This paper will contribute to a deeper understanding of obesity-related stigma in presenting the deeply embodied experiences of those living with obesity by using in-depth individual interviews with participants and photographs contributed by study members as they recount instances of obesity-related stigma throughout their lives.

### Materials and methods

Despite obesity-related stigma having been researched in prior studies (Mensinger et al., 2018; Phelan et al., 2015; R. M. Puhl & Brownell, 2003), exploring nuances of the lived experiences of obesity-related stigma in an Irish context can continue to add to understanding the phenomenon. The aim of this study was to explore the lived experience of those living with obesity as they experience obesity-related stigma. This study explored whether lived experience of obesity-related stigma was mediated through treatment outcomes/challenges. This is important to explore to fully understand how such experiences can compound the psychosocial impact of treatment-related outcomes/challenges on those living with obesity. This paper reports on two qualitative approaches adopted to explore the aim of the research, including individual conversational interviews and photovoice method. As indicated in the COREQ checklist it was ensured that details regarding methodological orientation, participant selection, data collection and data analysis were included in this paper (Tong et al., 2007).

### Conversational interviews

Conversational interviews were conducted to allow for an open and non-directive approach to explore the lived experience of people with obesity. The main characteristic of the conversational/hermeneutic interview is that only one very broad question is asked at the beginning of the interview. This question focuses on the phenomenon which is being explored (Van Manen, 1990). In doing so, the researcher ensures that the interest of the conversation will not change and that the conversation centres around the lived experience which is being investigated (Van Manen, 1990). The interviews were held online using Zoom. The research question posed for the purpose of this study was “can you tell me about your experience of living with obesity”. The researcher (EH or EF) assured to follow the hermeneutic approach by allowing the participant to guide the conversation (Van Manen, 1990). Thus, the researcher encouraged the interviewee to share their story without interruption. The interviewer would only ask questions during the conversational interview if she felt the need for clarification, to show understanding by repeating what the participant said or to ask about their feelings regarding a mentioned incident (Farrell et al., 2022). The interviews took between 45 and 105 minutes.

### Photovoice method

As the aim of the research project was to understand the phenomenon “living with obesity” from a participant’s perspective, the photovoice method (Luttrell, 2010) offered an opportunity for the researchers to gain visual insights into the interviewee’s concerns and priorities (Wang & Burris, 1997).

According to Luttrell (2010) the photovoice method can be used to change the narrative around people’s perspective on a specific phenomenon which they encounter. The participatory aspect enables researchers to learn about people’s lived experiences. This is because the participants are in charge and in control of what they photograph (Wang & Burris, 1997). According to Nykiforuk et al. (2011) it is recommended to set a limit to the number of pictures that participants are asked to take when applying the photovoice method. The authors also suggested to give participants enough time during the photovoice interview in which individuals are asked to explain why a photograph was taken, to learn about the participants’ full experience of capturing their lived experiences using a camera (Nykiforuk et al., 2011).

The researchers (EH, EF or DMcG) adapted this method (Luttrell, 2010; Nykiforuk et al., 2011; Wang & Burris, 1997) by asking participants to portray their concerns regarding obesity as well as their hopes and desires when engaging in obesity treatment and their experiences of living with obesity during Covid-19. Disposable cameras with a participants guide were posted to the interviewees by mail. They were given a timeframe to return those cameras (1 week) and an envelope was provided in which those cameras could be sent back. They were asked to take photographs to answer the following three prompts:

- 10 photographs to represent their concerns about living with obesity
- 10 photographs to represent their hopes and desires from engaging with treatment
- 5 photographs to represent their experience of living with obesity during Covid-10

The pictures were then developed, and individual interviews were conducted with the participants who were invited to tell the story of their photograph. After 2 hours a second interview was scheduled with the participant. At the end of each interview the participant was asked to choose five photographs to represent their experiences of 1. concerns of living with obesity, 2. hopes and desires when engaging with obesity treatment and 3. experience of living with obesity during Covid-19.

Twelve individuals participated in the photovoice activity, eight women and four men. Participants were offered the possibility to participate in audiencing interviews following the photovoice interview.

As a last step two “audiencing” interviews were conducted online with four participants (two participants per interview) (Luttrell, 2010, p. 228) in order for participants to share their stories behind the chosen photographs and to evoke a reflective discourse between the interviewees. The purpose of the audiencing interviews was to bring the participants together, share their photographs and to create a space to further explore the themes. Nykiforuk et al. (2011) described this step of the photovoice method as showing the study findings to other participants in a focus group interview to receive “further community feedback prior to broader community dissemination” (p. 120). Thus, participants were invited to a group interview to share and discuss their photographs with other participants (Luttrell, 2010).

All interviews and data collection activities were conducted online via Zoom between February and November 2021. The interviews were held online and not in person to ensure adherence to social distancing COVID-19 guidelines at the time (Government of Ireland, 2020).

Eligible candidates were contacted by the researcher via phone and provided with brief overview of the study. A follow-up email then provided to interested and potential participants with further information on the study through an information leaflet and a consent form. Individuals were invited to return their consent form via email if they wished to participate in the research and were informed that they could stop their participation at any point without consequence. Written consent was obtained for the individual conversational interviews, the photovoice methodology and the audiencing interviews. UCD’s Human Research Ethics Committee granted ethical approval for this study (HS-20-12-McGillicuddy).

### Sample

The researcher applied purposeful sampling to gain a deeper understanding for information-rich cases. This sampling technique strives to explore a phenomenon in-depth as opposed to drawing empirical generalizations (Patton, 2002). Eligible candidates (adults over the age of 18, with a BMI greater than 30 kg/m2) were identified via two Irish Clinical Research Centers which focus on metabolic diseases and weight management. Participants were divided into five subgroups as part of the recruitment to reflect the varying treatments being offered including lifestyle changes, pharmacotherapy,^2^ and bariatric surgery. Additionally, the team recruited individuals who are living with Type 1 Diabetes Mellitus and with Type 2 Diabetes Mellitus. Fifteen participants were recruited in total of whom eight were female and seven were male.

### Data analysis

An overview of participant characteristics is presented in Table I. The average age of participants was 56.9 years of age.

**Table I.** Participants’ pseudonyms, subgroups and characteristics.

Pseudonyms	Subgroup	Gender	Age
Angela	Bariatric Surgery (BS) (a week before the conversational interview)	Female	Chose not to disclose
Áine	Pharmacotherapy (PT)	Female	69
April	Type 1 Diabetes (T1D)	Female	Chose not to disclose
Ada	Pharmacotherapy (PT)	Female	47
Cora	Lifestyletherapy (LT)	Female	60
Catherine	Lifestyletherapy (LT)	Female	56
Daniel	Bariatric Surgery (BS) (close to surgery)	Male	Chose not to disclose
Freida	Bariatric Surgery (BS) (several years ago)	Female	55
Kenny	Pharmacotherapy (PT)	Male	69
Keith	Type 2 Diabetes (T2D)	Male	55
Liam	Bariatric Surgery (BS) (four months prior to the conversational interview)	Male	Chose not to disclose
Miriam	Type 2 Diabetes (T2D)	Female	50
Peadar	Type 2 Diabetes (T2D)	Male	56
Rory	Type 1 Diabetes (T1D)	Male	Chose not to disclose
Steve	Lifestyletherapy (LT)	Male	52

Each participant was assigned a pseudonym to protect anonymity. The interviews were recorded and transcribed verbatim. The transcribed interviews were analysed using the software NVivo. The data were first analysed to identify big overarching themes which emerged during the interviews. Obesity-related stigma revealed as an overarching theme from this first step of the analysis. This enabled the researcher to analyse the data in consideration of the research aim concerning the exploration of people’s lived experiences with obesity-related stigma. According to Braun and Clarke’s (2006) guidelines for thematic analysis, data from stigma were interpreted by separating data into paragraphs and identifying codes. Those codes were clustered. In a next step overarching themes were created based on the clustered codes. By reviewing the data a second time, the author adjusted the existing themes and added missing themes, to ensure that those themes represented the participants’ stories. The themes and subthemes pertaining to obesity-related stigma are presented in Table II below (Braun & Clarke, 2006). The photographs have been identified as part of data analysis of (a) conversational interviews and (b) photovoice method. The photographs were not identified as themes, rather they sit under each theme.

**Table II.** Themes.

**Theme** Subthemes	**1. Exposure to external judgement** 1.1 Judgemental comments 1.2 Hypervigilance to societal judgement
**Theme** Subthemes	**2. Societal exclusion** 2.1 Feeling overlooked and ignored 2.2 Effects of societal exclusion
**Themes** Subthemes	**3. Felt environmental stigmatization** 3.1 Felt environmental stigmatization when engaging in hobbies 3.2 Felt environmental stigmatization in everyday life

## Results

Evident from data analysis was the impact of obesity-related stigma on the lives of those participating in the study. This paper will present key findings capturing participants lived experience of obesity-related stigma under the following developed themes:

1. Exposure to external judgement

(1.1) Judgemental comments
(1.2) Hypervigilance to societal judgement

2. Societal exclusion

(2.1) Feeling overlooked and ignored
(2.2) Effects of societal exclusion

3. Felt environmental stigmatization

(3.1) Felt environmental stigmatization when engaging in hobbies
(3.2) Felt environmental stigmatization in everyday life

### Exposure to external judgement

#### Judgemental comments

Data from this study identified that the experience of obesity-related stigma was strongly linked to the exposure to external judgement. Participants reported perceived societal judgement based on their physical appearance and initial impressions made of them. These external judgements would be expressed using language perceived as hurtful such as lazy”, “fat^3^”, “ugly”, “stupid” and “slob” and endured since childhood. Those comments led to beliefs about being different from people in smaller bodies, as Miriam (T2D^4^) explained, who reported experiencing stigmatizing comments from people in smaller bodies feeling a sense of needing “conform into the average person to be with the small waistline and the perfect figure […] if you look anyway different at all that it’s very easy to get negative comments” (Miriam, T2D).

Stigmatising behaviour was also encountered when accessing weight loss clubs or when engaging with weight loss experts:

She [weight loss expert] says, “You’re not doing enough, you’re just not doing enough, you need to go out walking, you need to pound the streets”, I [Freida, BS] said, “I can’t, here I am, I’m on two crutches, I’m waiting for back surgery, I’m losing weight to get back surgery”, and she [weight loss expert] said, “You need to find a way to pound the streets”. (Freida, BS).

Participants also reported experiencing instances of external judgement in public spaces such as kindergarten, the workplace, in the media, the doctor’s office and from individuals including dieticians, friends, family, (potential) partners and the fashion industry. Ada (PT) experienced friends commenting on her weight gain during puberty and expressing shock about it. Comments such as “what happened”, “[you] used to be so pretty” (Ada, PT) impacted negatively on her well-being. Ada (PT) also reported of having received comments from family. A family member, who she and another person picked up from the hospital, insisted on sitting in the back seat of the car to avoid Ada (PT) having “to squeeze into the back”, which made her feel very uncomfortable. She reported standing up for herself as she told the family member “You know, I know I’m fat but I can still get back in there, is that okay with you?” (Ada, PT). Ada (PT) explained how this experience illustrated society’s perception of people with obesity whereby they “just see this as not acceptable in some ways, it doesn’t fit in and it’s not healthy, it doesn’t look good. […] it’s not sexy.” (Ada, PT).

Áine (PT) contributed a photograph from a fashion magazine showing an outfit which she would love to wear (Photograph 1) as “that’s the way I want to dress” (Áine, PT).

**Figure:**
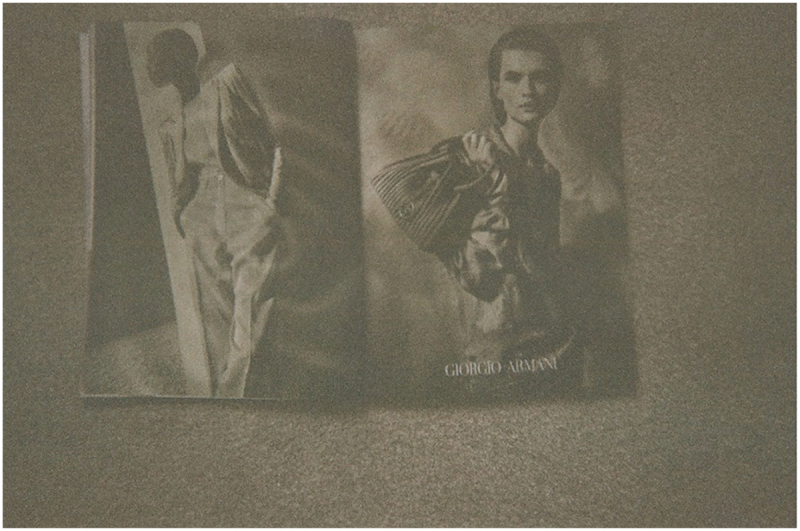
Photograph 1: Designer outfits (Áine, PT).

##### Photograph 1

Áine (PT) wished she would fit into the designer outfit in the photograph above (Photograph 1) but “he [designer] does not make for heavy people because the whole line of his design is lost if you have a curve you know so but [she] just love[s] his stuff so that was all, that’s just wishful thinking” (Áine, PT).

Some participants ascribed positive effects to exposure to external judgement. Rory (T1D) suggested that obesity-related external stigma might elicit a change of behaviour for people with obesity. He explained that being inhibited from participating in certain activities such as rollercoaster rides might result in motivation to manage one’s obesity whereby if “I couldn’t get on a ride because I was too big, I think that is a positive thing because it might give you a kick in the butt you need” (Rory, T1D). Explaining that tough love from his General Practitioner helped him to take the diagnosis seriously, he elaborated that other people might benefit from “the hard truths” (Rory, T1D) as well. Peadar (T2D) shared a similar experience during the audiencing interview as he reported that it is important to “own” obesity and “be tough on yourself”. Catherine (LT) agreed as she explained that there will be no other person but oneself to help manage obesity as “there’s nobody going to come and do it for you, you know? Or can’t, you know you have to take control and do it for yourself, you know?” (Catherine, LT).

#### Hypervigilance to societal judgement

Exposure to societal judgement resulted in hypervigilance to societal judgement. This was linked to participants’ perception of others constantly observing their behaviour and led to increased self-consciousness, as experienced by Freida (BS). Freida (BS) described how she and her sister had been publicly harassed for enjoying an ice cream on a warm day (Photograph 2).

**Figure:**
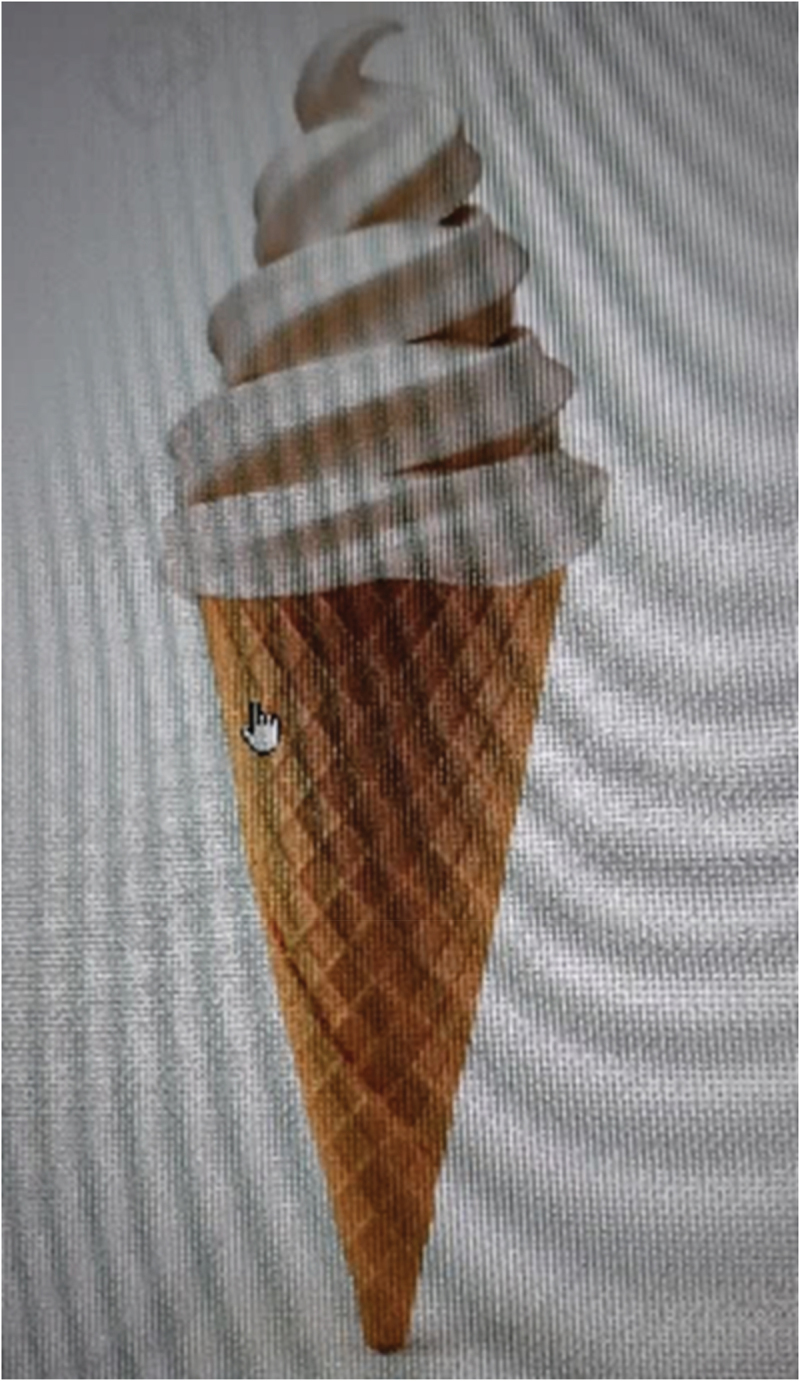
Photograph 2: The ice cream (Freida, BS).

##### Photograph 2

Freida (BS) explained that a woman continuously screamed at and insulted them, resulting in extreme discomfort and public humiliation as:

Well this woman came after us and she tackled us both for eating ice cream and the size of us and do we realise the damage we’re doing and I just said […] “We’re just having a hard day”, or something like that and she just kept coming at us and we got up and we walked away and she came after us and she was shouting after us and the size of you and you’ll get cancer and you’ll get heart disease and do you realise the cost on the State and I just kept walking and I said to [sister’s name], “Come on”, […] She was like shouting after us and we just went out to the car […] lucky enough we were together because if I had were on my own I probably would have gotten in to the car and cried. (Freida, BS)

When asked by another participant during the audiencing interview if she ever had an ice cream in public again she responded: “certainly not in public” (Freida, BS), illustrating how this experience resulted in her being hypervigilant towards societal judgement.

This overt and extremely public obesity-related stigma had a profound impact on Freida’s (BS) intentions regarding her interaction and behaviour in public in the future, essentially limiting her freedom to be in her life. Using a very powerful analogy, Freida (BS) explained how she experienced public stigma and exposure to judgement by comparing herself and others who are living with obesity to a mink which is trapped in a waggon pipe:

I used to say that I would [feel] like fur things […] like a mink, down a wagon pipe. Big white pipe, tall pipe and the pipe was filled with like grease or something and I get up a little and I’d fall back down, and I get over another bit and I fall back down and that’s the way every day was what’s happening to these people. They just can’t they put their head above the parapet, people push them down and that all. That stigma has to stop. (Freida, BS)

Experiences of hypervigilance to societal judgement were particularly strong in the grocery store, on holidays, when sea swimming, in the gym or swimming pool (Photograph 3).

**Figure:**
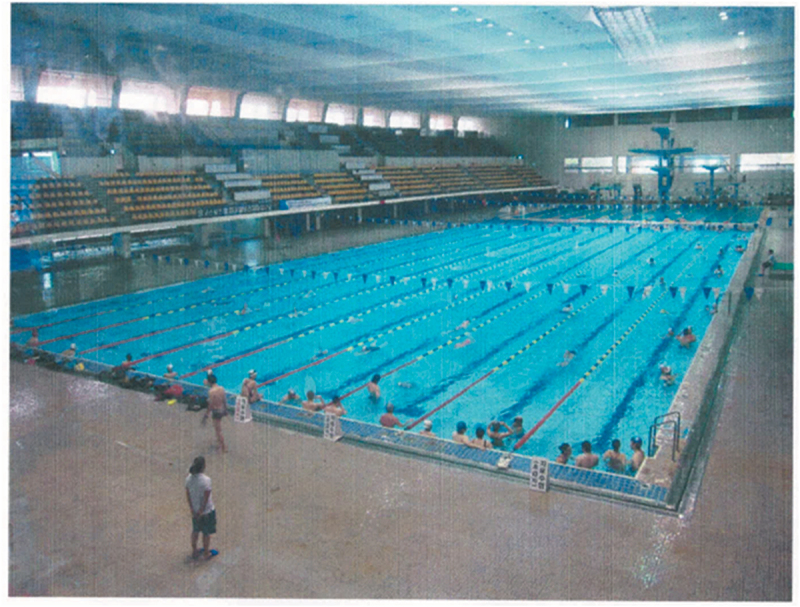
Photograph 3: Public swimming pool (Catherine, LT).

##### Photograph 3

Catherine (LT) elaborated that sport clubs were associated with athletic people and that she felt hypervigilant concerning the understanding that “you’re anything but that in a swimsuit” (Catherine, LT). Hypervigilance to societal judgement led to avoidance of exercise “in a public space” was, as shared by Miriam (T2D). A photograph of a garden door representing her journey of living with obesity reminded Miriam (T2D) of this experience (Photograph 4).

**Figure:**
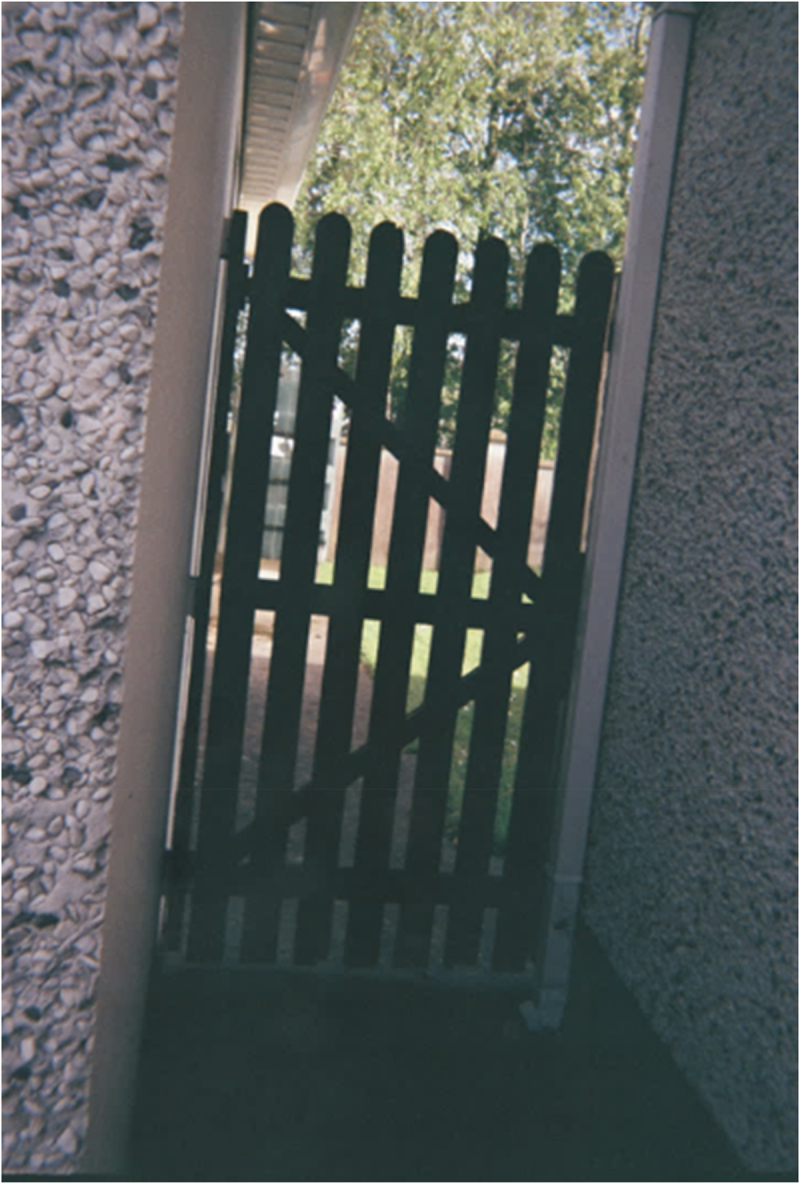
Photograph 4: The garden door (Miriam, T2D).

##### Photograph 4

Realising that being able to exercise helped Miriam (T2D) to get to the sunny side behind the door evoked memories of feeling exposed when doing sports as:

Exercise for me was the issue and I was very overweight, because you get to a point where it’s a real struggle to exercise […] it’s finding the right clothes to wear when you’re exercising because […] doesn’t look good on someone that big normally everybody else is going around and very tight gear […] I suppose it’s finding clothes as well you’re comfortable exercising in you don’t mind people. (Miriam, T2D)

Hypervigilance to societal judgement was also reported when going to the spa. Catherine (LT) explained that she felt similarly uncomfortable when accessing a spa facility (Photograph 5). Catherine (LT) explained that “it’s just not the image. You don’t see obese^5^ women walking around in white fluffy bathrobes that don’t close on them. So, it’s not the image in a spa. It really isn’t” (Catherine, LT).

**Figure:**
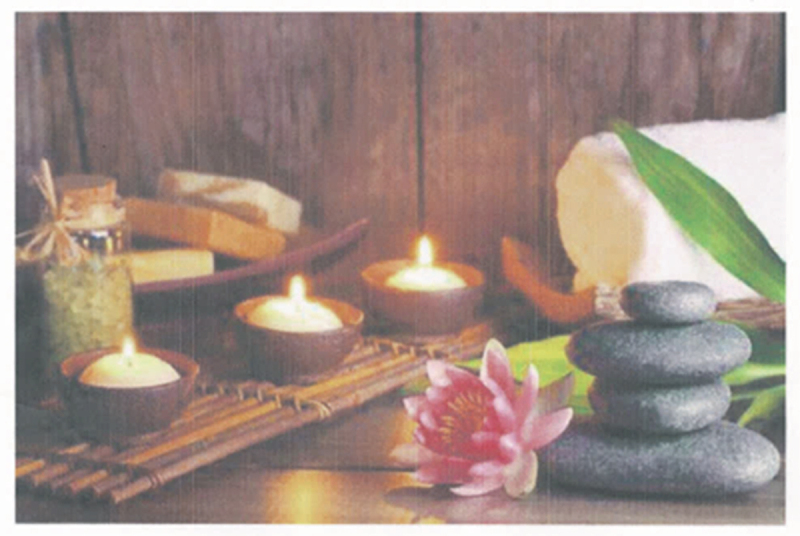
Photograph 5: Going to the spa (Catherine, LT).

##### Photograph 5

Participants reported that exposure to societal judgement from friends, family and strangers in public compounded feelings of being unwanted and excluded from society. Vocabulary used by participants in relation to exposure to societal judgement is presented in Table III. Awareness of societal judgement impacted on daily life as it inhibited people living with obesity from moving freely in public space. The lack of acceptance by others further compounded experiences of such exclusion.

**Table III.** Vocabulary used in relation to exposure to external judgement.

Exposure to external judgement
fat,^6^ ugly, stupid (Ada, PT)
lazy, slob (April, T1D)
“do you have a baby in there” (comment received from a child) (Cora, LT)
’oh, you put on so much weight’ or “I don’t recognize you” (Miriam, T2D)
[your] bum wiggles (Catherine, LT)
“The last thing I need now is for some fat^7^ woman to win this outfit” […] “Because it’s just not going to look good” (comment made by a designer) (Áine, PT)
was way too overweight and she’d never look well no matter how good her voice was, she wouldn’t look well’ (comment made by a designer) (Áine, PT)
just looked at [her], and [she] know[s] by him I disgusted him (stare received by a doctor) (Freida, BS)
you have to lose weight before I touch you (comments made by a doctor) (Freida, BS)
well you know you’re just too fat^8^ (comments made by doctor’s colleague) (Freida, BS)
what you need to do is pushbacks’, and I said, ‘What are pushbacks? “Push back from the table” (comment received from doctor) (Freida, BS)
all I can do is for you to lose your weight is to wire your mouth together (comment received from doctor) (Angela, BS)
she’d have to move in with me to check what I was doing, I thought, she doesn’t believe it (comments received form dietician) (Áine, PT)
I’m surprised at you and you’re so careful about your appearance to let yourself become like that (comment received from a doctor) (Peadar, T2D)
“Oh fattie,^9^ don’t come near me because you’re high risk” (joke made in the media during Covid) (Ada, PT)
what happened, [you] used to be so pretty (comment received from friends) (Ada, PT)
“Look, if you lose weight I will pay for your ticket” (comment received from a family member) (Ada, PT)
you’re a bit big for that (comment received from a family member) (April, T1D)
you’re putting on too much weight (comment received from family member) (Keith, T2D)
“We’re going home to my house for dinner”, I always remember he said, “But don’t worry I told mum you were on a diet” (received comment from partner) (Áine, PT)
“[Other person is] just looking at me now and she’s totally disgusted with what she sees” (Freida, BS)
“Oh, [Ada] is a really nice girl, but her body just doesn’t fit onto my motorbike” (someone she was romantically interested in) (Ada, PT)
saying look what’s in her trolley. You know, look what’s in her trolley, she’s got crips no wonder, she is the size she is (Freida, BS)
she tackled us both for eating ice cream and the size of us and do we realize the damage we’re doing and I just said […] “We’re just having a hard day”, or something like that and she just kept coming at us and we got up and we walked away and she came after us and she was shouting after us and the size of you and you’ll get cancer and you’ll get heart disease and do you realize the cost on the State (Freida, BS)

### Societal exclusion

#### Feeling overlooked and ignored

Participants linked living with obesity and obesity-related stigma to being excluded from society. Miriam (T2D) explained that she felt “invisible” in public due to living with obesity. She described how others did not notice her when running daily errands and that she felt that people were ignoring her whereby she felt that “when I went into a shop or, um I went around the town, I felt because of my size that people didn’t really see me and if they did see me they didn’t like what they’ve seen” (Miriam, T2D). Being excluded from Christmas events was also reported by Miriam (T2D), as she mentioned that her partner at the time would not invite her as “I remember not being asked to Christmas parties […] and I used to feel, even though he’d never say it, I used to feel it was because of my size” (Miriam, T2D). Áine (PT) shared a similar experience as she mentioned that due to living with obesity people did not notice her “because you’re just part of the blobby people, just that crowd over there you know whereas you don’t stand out for any reason” (Áine, PT).

Angela (BS) described how COVID-19 contributed to this sense of exclusion. Being assigned to the at-risk category exacerbated feelings of isolation as “the relationship between Covid um the vaccine is a societal and policies at the moment are segregating it’s not your choice of isolation, it’s just the systematic isolation” (Angela, BS). She shared another very powerful statement of society’s behaviour as “society punishes the person for the overweight” by isolation (Angela, BS). A lack of societal acceptance concerning people with obesity resulted in individuals being punished by being isolated from societal “joy”, “celebration” and “participation” (Angela, BS). Miriam (T2D) explained that individuals who are living with obesity are “not seen as the same value” and “treated as second class citizens” (Miriam, T2D).

#### Effects of societal exclusion

Participants reported various effects of being excluded from society. Catherine (LT) described that she felt “excluded from what other people would just take as the norm” (Catherine, LT). Miriam (T2D) mentioned during the audiencing interview that being “overlooked” can result in people with obesity feeling lonely and pretending to be someone else (Photograph 6).

**Figure:**
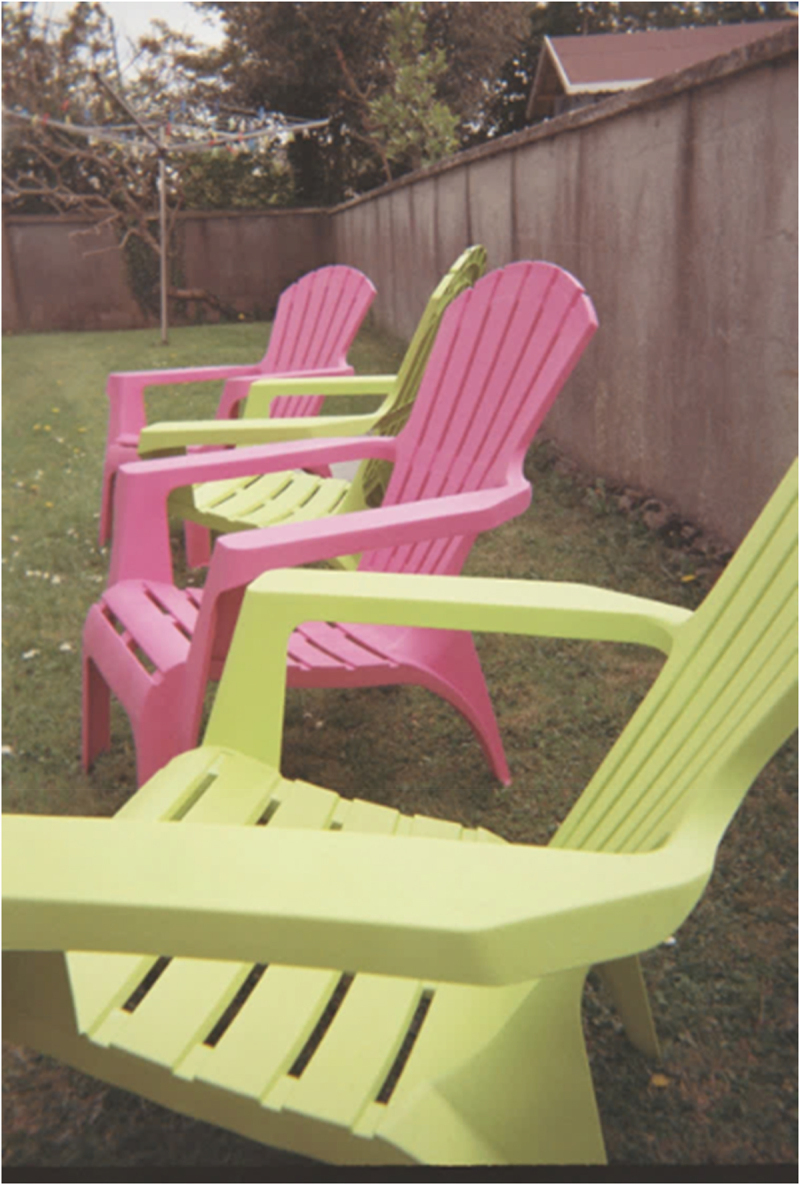
Photograph 6: Empty chairs (Miriam, T2D).

##### Photograph 6

A photograph of empty chairs made her think about this consequence of being excluded as she explained that:

The chairs, you know, in a way, maybe represent a loneliness, I suppose, you know, the fact that are empty. […] obesity can be a very lonely place to be for a lot of people […] where people are overlooked and lost. […] I definitely feel that, when I was in that place, I was, felt very lost, […] and it takes a while to find yourself again, so I suppose yeah you could, you could say that, they would could also represent a loneliness that you go through as an obese^10^ person and even though you might be a co- a larger than life character […] when compared to society because you’ll find that a lot of obese people take on this persona of this big character […] and be able to laugh at themselves but inside I think they’re quite lonely and quite lost and you know it, it’s hard to reach them. (Miriam, T2D) (Photograph 6)

Angela (BS) referred to “the norm in society” when talking about dress codes at work. She explained that people with obesity are excluded from the norm of wearing tight leggings or bodysuit to work, which made her feel very uncomfortable as:

In the workplace or you see everybody our working now with leggings and these real tight Lycra bodysuits, you know, they don’t leave anything to the imagination. And you’re there thinking God, you know, there’s no way I’m going to go near that, do you know. (Angela, BS)

Losing weight in order to fit into society has resulted in experiencing “more talking about food, it creates an obsession with food.” (Angela, BS). She also described that she felt that there were no categories of gyms where people with obesity fitted in as she explained that “gyms are opening up everywhere, do you know […] and for males particularly and females, it can be, do you know, if you don’t fit into that category, then, where do you fit in?” (Angela, BS). Ada (PT) shared a similar thought, whereby society is forcing people who are different from others “into the normality” (Ada, PT). This perceived active societal exclusion was reflected in the perception that “you’re not the type of people that they want in society” (Miriam, T2D).

Obesity-related stigma manifested in individuals feeling ignored and underrepresented in the public. This exacerbated experiences of being othered. Vocabulary used by participants in relation to societal exclusion is presented in Table IV. Similar to exposure to external judgement, societal exclusion was linked to a lack of acceptance of obesity in public spaces. Scarcity of suitable equipment for people with obesity compounded experiences of public stigma.

**Table IV.** Vocabulary used in relation to societal exclusion.

Societal exclusion
invisible (Miriam, T2D)
because you’re just part of the blobby people (Áine, PT)
Covid um the vaccine is a societal and policies at the moment are segregating it’s not your choice of isolation, it’s just the systematic isolation (Angela, BS)
not seen as the same value, treated as second class citizens (Miriam, T2D)
excluded from what other people would just take as the norm (Catherine, LT)
overlooked and lost (Miriam, T2D)
gyms are opening up everywhere, do you know […] and for males particularly and females, it can be, do you know, if you don’t fit into that category, then, where do you fit in (Angela, BS)
you’re not the type of people that they want in society (Miriam, T2D)

### Felt environmental stigmatization

#### Felt environmental stigmatization when engaging in hobbies

Individuals reported experiences whereby felt environmental stigmatization led to experienced obesity-related stigma. This included their experiences in attempting to access resources and equipment in daily life. Participants in this study reported a lack of access to suitable equipment, and at times, such resources were missing. Having to request equipment or resources required to support their needs was often associated with feelings of being stigmatized in public. One example of felt environmental stigmatization negatively impacting on the lives of those living with obesityrelated to engaging in hobbies. April (T1D) mentioned that the experience of being assigned a “particularly big horse” during horseback riding class due to her weight was “mortifying” (Photograph 7).

**Figure:**
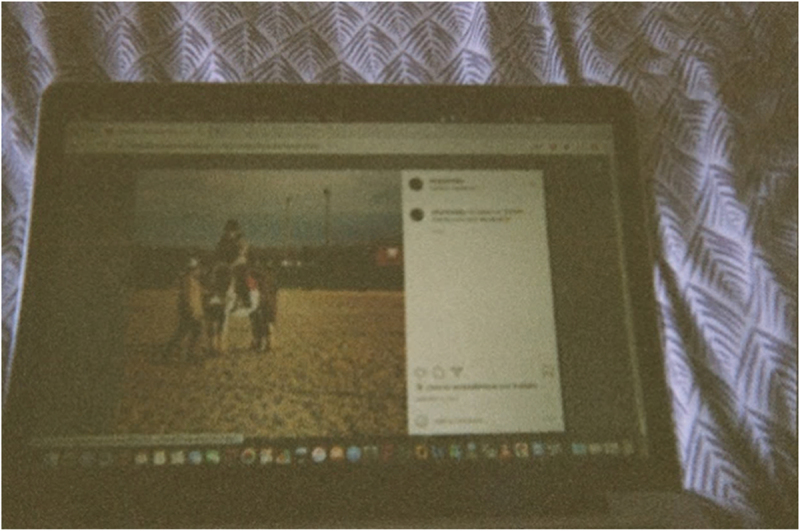
Photograph 7: Horseback riding (April, T1D).

##### Photograph 7

April (T1D) explained how the instructor announcing the need for a bigger horse in front of the others made her particularly uncomfortable, whereby:

[She] was mortified going in on the first day, “How much do you weigh?” And [she] said, “You know and I kind of real ashamed you know I weight about 18/19 stone”, “Right okay, yeah no you won’t be able to go on him” and she [the instructor] calls over to one of the other girls in the stable, “Actually will you get [name of the horse] out for me, this lad’s too small”.

(April, T1D)

One’s ability to partake in fun activities being impacted by unsuitable equipment was also described by Catherine (LT) (Photograph 8).

**Figure:**
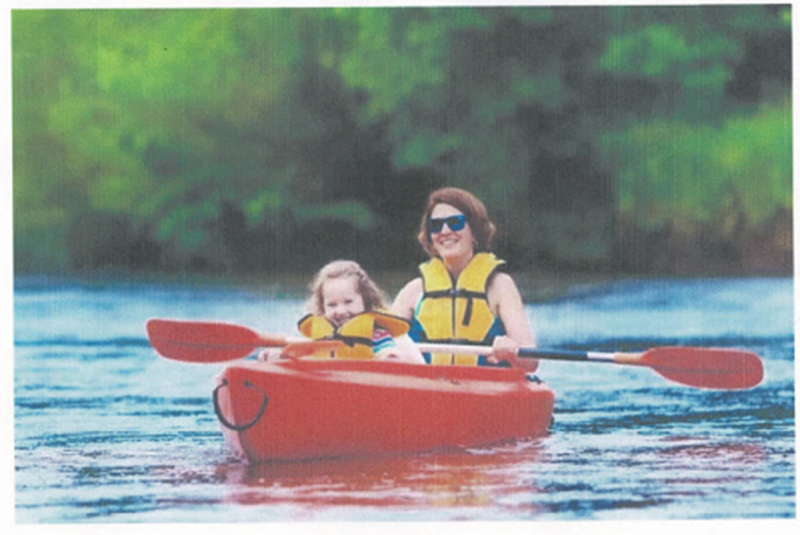
Photograph 8: Sense of adventure (Catherine, LT).

##### Photograph 8

Being a fan of outdoor water sport activities herself (Photograph 8), she explained that concerns about the availability of a suitable lifejacket and kayak for people with obesity would keep her from enjoying such activities explaining that photograph 8 represented:

That one is just the thrill of going out. […] And then actually again you’ve got your lifejacket size. The strength to canoe, to literally paddle your own canoe or kayak or whatever and just the thrill of being out in the open air with the grandchild. That all appeals to me. It’s kind of the good and the bad. The joy of it but would you fit into a kayak when you’re overweight? (Catherine, LT)

#### Felt environmental stigmatization in everyday life

Experiencing stigma due to unsuitable, missing or larger sized equipment impacted how participants experienced everyday activities and social events, such as going to the cinema, theatre, restaurant, rollercoasters, fun activities on holidays or using a car, public transport and airplane. As Peadar’s (T2D) described “[he] remember going to Gardaland, near Lake Garda, and I kind of more or less had to sit out on a good few things, because they just, they’re not made for guys my size” (Peadar, T2D). Being asked to choose the plus size options when shopping online for clothes was also perceived as stigmatizing. Ada (PT) reported that, while feeling relieved about the availability of a bigger variety of fitting clothes, she also mentioned that clicking on the plus size button made her feel self-conscious as:

It’s definitely got better with clothes, you know there’s more choice and everything, but you still the fact, you know that you have to go to a page, and that is specifically after click on you know for plus size, you know and then all of that and it’s you know you can’t go to a normal shop. (Ada, PT)

Missing accessibility in the workplace and a lack of space in the work environment led to feelings of discrimination. Concerns regarding the lack of space in car parks, Catherine (LT) mentioned that “it would be nice if the parking was standard bigger size” (Catherine, LT) (Photograph 9). Comparing Ireland to the United States of America, Catherine (LT) pointed out that “all the infrastructure is there and allowance or accommodations or whatever for larger people” (Catherine, LT).

**Figure:**
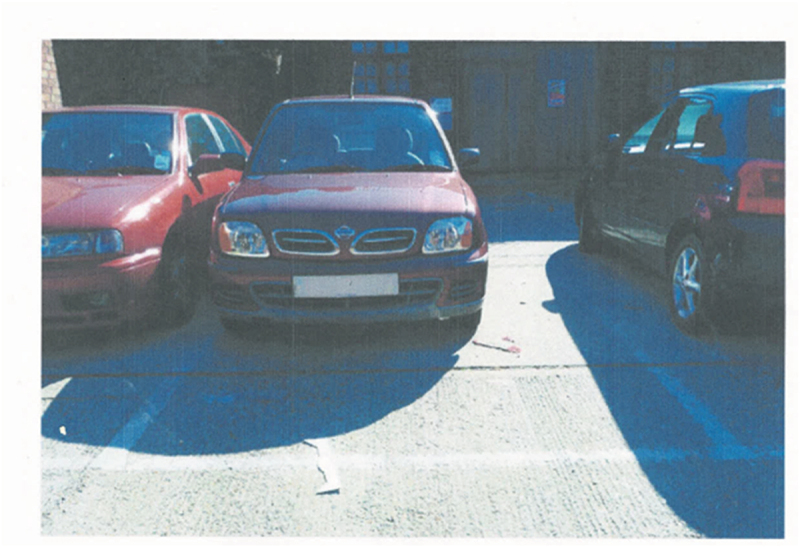
Photograph 9: Parking concerns (Catherine, LT).

##### Photograph 9

Interestingly, Rory (T1D) shared a contrasting experience as “Ireland is going that way as well, it’s not as extreme as the States but it’s a lot easier to be overweight now than it was say 20 years ago” (Rory, T1D), arguing that people with obesity will not manage their obesity unless there is “a need to change” (Rory, T1D).

Participants reported the felt stigmatizing effects of accessing equipment and resources within the built environment, the associated visibility this brought to their lived experience of living with obesity and the embodiment of shame arising from the unwanted attention. Such experiences were deeply embodied within the psyche of those living with obesity, shaping how they interacted with the world around them and hence impacting on their everyday lives in the public and private spaces within society. Vocabulary used by participants in relation to felt environmental stigmatization is presented in Table 5.

**Table V.** Vocabulary used in relation to felt environmental stigmatization.

Felt environmental stigmatization
calls over to one of the other girls in the stable, “Actually will you get [name of the horse] out for me, this lad’s too small”, mortifying because they had to like get a particularly big horse for me (April, T1D)
no, actually we don’t have your size (Catherine, LT)
let’s be honest. You don’t see very large women or man in wetsuits going diving. Again, not the normal image. (Catherine. LT)
push their chair in to allow you pass, that sort of, that you’re inconveniencing somebody (Catherine, LT)
taking up maybe two or three spaces where there’s one person in another space (Miriam, T2D)
conspicuous, stand out (Miriam, T2D)
they’re not made for guys my size (Peadar, T2D)
thinking “Yeah, I can’t fit in that” (Catherine, LT)

## Discussion

This study explored the lived experience of obesity-related stigma. Previous research has identified the profound negative impact of obesity-related external stigma on physical (Jackson, 2016) and mental wellbeing (Alimoradi et al., 2020; Jackson, 2016) and the negative effects of obesity-related stigma on the quality of life of people living with obesity and on leading a healthier lifestyle and the reduction or maintenance of body weight (R. Puhl & Suh, 2015). The following three themes were developed from this qualitative study of obesity-related stigma: exposure to external judgement, societal exclusion and felt environmental stigmatization.

Evident from the data was that obesity-related stigma as exposure to external judgement was experienced throughout participants’ lives and profoundly impacted them. Obesity-related stigma manifested through feeling surveilled in public in the form of hurtful comments based on other’s first visual impression of people with obesity, which supports findings from Vartanian et al. (2014) and Lewis et al. (2011). Particularly profound was the language that was used by others to express their judgement about people with obesity, by using vocabulary such as fat, ugly, stupid, lazy and slob.

Stigmatising behaviour was experienced from family, friends, potential partners, media, fashion industry, healthcare professionals, dieticians, weight loss clubs and experts. Particularly striking were findings from the study regarding the experience of feeling different from other people, as the average person was perceived to live in a smaller body. This resonates with findings from Ueland et al. (2019), as the authors suggest that participants of their study “experienced living with obesity as an objectification and alienation from themselves and the world” (p. 9). There is a correlation between the above mentioned results and Farrell et al. (2021) qualitative synthesis, which identified stigma, judgement, blame and shame as fundamental lived experience of people with obesity.

In contrast, positive effects of stigma in public were also experienced, which were linked to an urge to change one’s behaviour due to the uncomfortable effects of other’s judgement and the perceived importance of taking responsibility for living with obesity. However, those perceptions contrast with findings of obesity-related stigma hindering the reduction of obesity prevalence (Flint, 2015), resulting in mental health issues such as depression (Alimoradi et al., 2020) and a negative impact on quality of life (R. Puhl & Suh, 2015).

It was evident from the study that compounding obesity-related stigma through societal exclusion encompassed being overlooked, ignored, isolated as well as punished and treated like a member of a different class by others. This had negative effects on the lives of those living with obesity, particularly on their sense of belonging, as they felt excluded, limiting their life experiences. Exclusion from society was experienced as feeling lonely, pressured to fit in juxtaposed with a feeling of being unwanted as a member of the broader community. This supports findings from a study conducted by Lewis et al. (2011) as social isolation was identified as one of the consequences of stigma. Those findings also resonate with results from Thomas et al. (2008) research, which identified discrimination and social isolation as lived experiences of people with obesity.

Similar to findings regarding compounding obesity-related stigma through societal exclusion, this study echoes what data from Lewis et al. (2011) research showed regarding environmental stigma, as findings from this study indicate that obesity-related stigma through felt environmental stigmatization was linked to experiences of a lack of suitable equipment and clothing. This felt experience was described as a concern of being discriminated due to the lack of suitable equipment. In contrast to exposure to external judgement and societal exclusion, which was reported as enacted stigma (Prunty et al., 2020; R. Puhl & Brownell, 2001; R. M. Puhl & Heuer, 2009; R. Puhl & Suh, 2015), felt environmental stigmatization was experienced as felt stigma (Scambler, 1998). This feeling was particularly strong when engaging in hobbies and social activities such as horseback riding, outdoor sports and clothes shopping. Small seats in airplanes, in cinemas, theatres, on rollercoaster rides or on means of public transport and missing equipment to accommodate people with obesity in the workplace and carparks were also experienced as contributing to stigma. This lack of suitable items restricted people with obesity in their everyday lives.

Most striking is the finding that participants in this study felt different and excluded from society as a result of living with obesity. Obesity-related stigma was experienced as societal exclusion from everyday life experiences, limiting who they are and how they engaged with those around them from family to strangers. Indeed, this feeling of surveillance further compounded obesity-related stigma, evoking a sense of hypervigilance among those living with obesity as they navigated the everyday experiences of life, such as going shopping, eating out or engaging in activities. This omnipresence of obesity-related stigma impeded people with obesity from participating fully in life which led to their lives being limited.

It is evident from this study that there is much work to be done in addressing obesity related stigma not only within the medical field, but across social, political, economic and cultural spheres of increasingly complex and diverse societies. Of particular note is the limiting impact of obesity-related stigma on the lives of those living with this disease, evoking deeply embodied psychosocial responses of shame, fear, and most powerful of all, exclusion. The “A Healthy Weight for Ireland” campaign set out an obesity policy and action plan for the years 2016–2025, which includes anti-stigma courses for teachers to reduce obesity-related stigma in Ireland’s schools (An Roinn Sláinte – Department of Health, 2016). The issue of obesity-related stigma has also been discussed in the Irish Times (Houston, 2022). An article was released in 2022 (Houston, 2022) which reported findings concerning obesity-related stigma in the Irish healthcare system from a study conducted by O’Donoghue et al. (2021). This public discourse shows the importance of tackling obesity-related stigma in society. Future policies should focus on reducing obesity-related stigma not just in schools but in other areas of public life as well, such as in healthcare and the workplace, etc. Indeed, if we are to translate this deeper understanding of the lived experience of obesity into meaningful change in policy and practice, then it is incumbent on those working within the field to challenge the compounding of stigma as reported in this study.

## Limitations of the study

Due to the small sample size, findings from this study lack generalizability. However, as this study was aiming to gain an in-depth understanding of people with obesity’s lived experiences of obesity-related stigma the number of participants (*n* = 15 in conversational interviews and *n* = 12 in photovoice method) helped to facilitate this. The small sample size helped the researcher to spend more time with the participants and learn about their experiences. The study is lacking diversity due to the ethnic homogeneity of the sample. Another limitation is that not every participant’s age was recorded. Participants’ BMI was not recorded as it was deemed as too sensitive.

## Suggestions for future research

Future research on the lived experience of obesity-related stigma with a bigger, more diverse study sample could allow for more in-depth analysis of the different experiences between subgroups such as age and ethnicity. Identifying how obesity-related stigma can be reduced in the workplace, fashion industry, media, public places (such as cinemas, public transport, etc.) by using qualitative research methods can help to create an understanding of how to tackle the issue. Future research focussing on socio-economic status in relation to obesity-related stigma could facilitate the understanding of the phenomenon from a broader perspective. Further work identifying how to challenge obesity-related stigma could be helpful in addressing the issue in the future.

## Conclusion

It is evident from the findings of this study that obesity-related stigma has a profound impact on the lives of those living with obesity, shaping how they engage in both the physical and socio-emotional aspects of their lives. Particularly stark was the way in which obesity-related stigma-shaped perceptions of being judged by others resulting in a reported sense of hypervigilance when engaging in the world in everyday life. The compounding of obesity-related stigma as experienced in and through judgement by family, friends and strangers worked to position people living with obesity at the margins of their lives. The chronic experiencing of obesity-related stigma in all aspects of everyday life, including judgement, exclusion and environmental stigmatization, evoked strong psychosocial responses of blame and shame with participants feeling excluded and othered from broader society. Such lived experiences are especially concerning given the increasing number of people living with obesity globally.

This perceived exclusion from society as defined through obesity-related stigma also contributed to the shrinking of the world for those living with obesity in this study. Indeed, participants referred to this as a “life-limited” as a direct result of being stigmatized as an individual living with obesity. While stigmatization was experienced through explicit and overt engagement with others in society, the more covert and unintentional experiences of obesity-related stigma through the physical environment was equally as negatively impactful on the psychosocial wellbeing of those living with obesity. Indeed, the everyday, passive, implicit and explicit experiences of obesity-related stigma evident within this study serve to further marginalize and shame those living with obesity, negatively impacting their psychosocial wellbeing and limiting their engagement in everyday life.

It can be argued from this study that the social injustice of being positioned as other at the margins of our societies as a direct result of living with obesity is perhaps one of the most socially acceptable forms of stigmatization within our societies. This is especially evident in the experiences of obesity-related name calling, the lack of accessibility within the built and physical environment, societal judgement as experienced through negative comments and harassment by strangers in public and, perhaps most hurtful of all, negative interaction with family and friends. The reported feelings evoked by such experiences included shame, loneliness, discomfort, invisibility, disliked, isolation, lost, mortified and self-conscious. The embodiment of these feeling in the form of self-stigmatization contribute further to the self-limiting of the lives of those living with obesity. If we are to free those living with obesity from the visible and invisible shackles limiting their lives through stigmatization, it is critical that we address the socially acceptable injustice associated with living with this chronic and complex disease.

## Notes

1. This project aims “to enable healthcare professionals to reliably predict the complications of obesity and who will respond to treatment” (IMI2 SOPHIA, n.d.., para 1). The project employs a consortium of 31 partners within Europe and the United States of America.

2. This study was undertaken prior to the release of Ozempic®. Participants in the pharmacotherapy subgroup received Liraglutide at some point prior to the study.

3. This is a direct quote from a participant, thus the term has not been changed.

4. The following abbreviations were used to describe participants’ assigned subgroups: LT-Lifestyletherapy, PT-Pharmacotherapy, BS-Bariatric Surgery, T1D-Type 1 Diabetes, T2D-Type 2 Diabetes.

5. This is a direct quote from a participant, thus the term has not been changed.

6. This is a direct quote from a participant, thus the term has not been changed.

7. This is a direct quote from a participant, thus the term has not been changed.

8. This is a direct quote from a participant, thus the term has not been changed.

9. This is a direct quote from a participant, thus the term has not been changed.

10. This is a direct quote from a participant, thus the term has not been changed.
